# Prevalence of somatic comorbidities among coronavirus disease 2019 patients in Croatia in the first pandemic wave: data from national public health databases

**DOI:** 10.3325/cmj.2020.61.518

**Published:** 2020-12

**Authors:** Krunoslav Capak, Ivana Brkić-Biloš, Verica Kralj, Tamara Poljičanin, Mario Šekerija, Pero Ivanko, Željka Draušnik, Sandra Mihel, Danijela Fuštin, Ivan Cerovečki, Marija Švajda, Jelena Dimnjaković, Gordan Sarajlić, Tomislav Benjak

**Affiliations:** 1 Croatian Institute of Public Health, Zagreb, Croatia; 2 University of Mostar School of Medicine, Mostar, Bosnia and Herzegovina; 3 Andrija Štampar School of Public Health, University of Zagreb School of Medicine, Zagreb, Croatia; 4 University of Rijeka School of Medicine, Rijeka, Croatia

## Abstract

**Aim:**

To determine the prevalence of common somatic comorbidities among coronavirus disease 2019 (COVID-19) positive patients in Croatia in the first pandemic wave, and assess the differences in clinical outcomes depending on the presence of comorbidities.

**Methods:**

We analyzed data from patients confirmed to be SARS-CoV-2-positive from February through May 2020. The data were obtained from clinical laboratories, primary health care providers, and hospitals. Previously recorded comorbidities, including diabetes, cancer, circulatory diseases, chronic pulmonary, and kidney disease, were analyzed.

**Results:**

Among 2249 patients, 46.0% were men (median age 51 years; median disease duration 27 days). Hospitalization was required for 41.8% patients, mechanical ventilation for 2.5%, while 4.7% of all patients died. Patients who died were significantly older (median 82 vs 50 years, *P* < 0.001) with a higher prevalence of all investigated comorbidities (all p’s <0.001), more frequently required mechanical ventilation (34% vs 1%, *P* < 0.001), and had shorter length of hospital stay (median 13 vs 27 days, *P* < 0.001) with no sex preponderance. Patients requiring mechanical ventilation were significantly older (median age 70 vs 51 years, *P* < 0.001), more frequently men (59.6% vs 45.7%, *P* = 0.037), showed a higher prevalence of all comorbidities except ischemic heart and chronic kidney disease (all p’s <0.001), and demonstrated a higher case-fatality rate (63.2% vs 3.2%, *P* < 0.001).

**Conclusion:**

COVID-19 patients who died in the first pandemic wave in Croatia were more likely to suffer previous somatic comorbidities. This corroborates the findings of similar studies and calls for further research into the underlying disease mechanisms, hence providing ground for more efficient preventive measures.

At the end of 2019, a novel severe acute respiratory syndrome coronavirus 2 (SARS-CoV-2), causing coronavirus disease 2019 (COVID-19), was detected in the Chinese province of Hubei. It rapidly evolved to a worldwide public health emergency (1). The first confirmed case of COVID-19 in Croatia was reported on February 25, 2020 and by May 31, 2020, there were 2249 laboratory-confirmed cases of COVID-19, with 106 deaths (2,3).

A Chinese nationwide research on the impact of comorbidities on clinical characteristics and prognosis revealed that symptomatic COVID-19 patients commonly had circulatory and endocrine comorbidities. Moreover, patients with at least one known comorbidity had worse clinical outcomes (4). Other studies reported that the most common comorbidities in COVID-19 patients were circulatory system disorders, followed by endocrine disorders (5-8).

A UK electronic record study analyzing almost 5700 deaths attributed to COVID-19 found that death from COVID-19 was strongly associated with male gender, older age and socio-economic deprivation, uncontrolled diabetes, severe asthma, and various other pre-existing medical conditions (9).

A meta-analysis of COVID-19 disease severity, encompassing almost 60 000 patients, showed that most cases were mild (81.4%), while 13.9% were severe and 4.7% were critical. The rate of patients requiring admission to an intensive care unit was comparatively low (8.3%), while the use of supplementary oxygen therapy (38.9%), non-invasive (7.1%) and invasive ventilation (28.7%), and even extracorporeal membrane oxygenation systems (0.9%) was surprisingly high. Because of oxygen dependency in this patient group, pharmacological and/or supportive intervention was documented, however, parameters of hypoxia, such as partial pressure of oxygen, oxygen saturation, or respiratory rate were not reported. Therefore, real disease severity could not be adequately determined, and the exact role of supplemental oxygen, whether as a therapeutic or preventive measure, remains unclear (10).

In a study of nearly 500 patients hospitalized for COVID-19, intubated patients had a significantly higher median age and diabetes rate as compared with non-intubated patients (11). Another study of 98 patients hospitalized for COVID-19 showed that significant risk factors for both death and the need for respiratory support were nosocomial COVID-19 acquisition, diabetes, chronic lung diseases, and chronic neurologic diseases (12).

The aim of our study was to determine which comorbidities affected patients who required ventilator respiratory support and patients who died of COVID-19 during the first pandemic wave (February-May 2020) in comparison with those patients who did not require ventilator respiratory support and those who survived.

## Participants and methods

Data from several different data sources, including clinical laboratories, primary health care providers, and hospitals were collected, combined, and analyzed. Data from clinical laboratories and primary health care providers were collected through the Central Health Information System of the Republic of Croatia (CEZIH), owned by the Ministry of Health of the Republic of Croatia. CEZIH data were submitted for analysis to the Croatian Institute of Public Health (CIPH) by the Croatian Health Insurance Fund (CHIF), which operates the central segment of the CEZIH system (13). SARS-CoV-2 test results from all individuals whose nasopharyngeal swab samples were collected in Croatia from January 30, 2020 to May 31 2020 were obtained from clinical laboratories; the type of testing used was SARS-CoV-2 real-time polymerase chain reaction. Furthermore, data pertaining to all COVID-19 patients' visits to their primary health care providers from January 1, 2018 to May 31, 2020 were collected. All diagnoses recorded during the visits were analyzed to determine the comorbidities of COVID-19 patients.

The source of data pertaining to hospital patients was the National Public Health Information System of Croatia (NAJS), administered by the CIPH. Public health registries and databases include several business domains of the NAJS system; information was obtained from databases in the hospital domain, ie, hospitalization, rehabilitation, and day-care hospital treatment database (14). Data in this database were obtained from in-patient statistical forms (JZ-BSO), completed whenever a patient is discharged from hospital. All health institutions involved in providing stationary health care are obliged to complete and submit JZ-BSO forms to the NAJS system using the provided data channels. We analyzed all JZ-BSO forms from the period from January 1, 2018 to May 31, 2020 available in the NAJS system containing information on COVID-19 patients. The variables of primary interest were the main diagnosis at discharge and other diagnoses recorded during hospital treatment. The purpose of this analysis, as with primary health care providers’ data analysis, was to determine the range of diagnoses, ie, comorbidities recorded in patients concurrently with the COVID-19 infection.

The data on COVID-19 patients requiring therapeutic ventilation support and patients who died of COVID-19 were collected directly from hospital reports, independently of the mentioned data sources. Ethical approval for this research was granted by the CIPH Ethics Committee (381-03-351-17-3).

Data analysis was conducted during June and July 2020 at CIPH. The data on COVID-19 patients and their comorbidities recorded in CEZIH and NAJS systems, as well as all other data relevant to the study, were extracted using SQL Server Management Studio 18 software (Microsoft, Redmond, WA, USA) and combined to a single patient comorbidity database. The inclusion criterion was the diagnosis of COVID-19 per World Health Organization International Statistical Classification of Diseases and Related Health Problems, 10th revision (ICD-10), code U07; all COVID-19 diagnoses were laboratory-confirmed. Individual comorbidities were identified by their ICD-10 codes as follows: cancer – C00-C97; diabetes mellitus – E10-E14; arterial hypertension – I10-I15; ischemic heart disease – I20-I25; cardiomyopathy – I42; cerebrovascular disease – I60-I69; diseases of the circulatory system excluding hypertension – I00-I09 and I20-I99; chronic lower respiratory diseases – J40-J47; other chronic obstructive pulmonary disease – J44; chronic kidney disease – N18. The inclusion criterion for diabetes mellitus and arterial hypertension was the presence of more than one entry pertaining to the respective condition in the CEZIH database. All other studied comorbidities were recorded if more than one respective entry was present in the CEZIH database or at least one entry was present in the NAJS database. Disease duration for hospitalized patients was calculated as the interval between the first SARS-CoV-2 positive test result and hospital discharge or death. For patients who did not require hospital treatment, disease duration was calculated as the interval between the first SARS-CoV-2 positive test result and the second consecutive SARS-CoV-2 negative test result or death. If two consecutive SARS-CoV-2 negative test results could not be obtained within 28 days from the first positive test result, the maximum disease duration was set at 28 days.

Possible duplicate or redundant entries were eliminated using a common identifier (health insurance identification number issued by CHIF). The normality of distribution was tested with the Shapiro-Wilks test. Differences between groups of independent continuous variables were analyzed with the Mann-Whitney U test, whereas differences in the prevalence of individual conditions were compared with the χ^2^ test and Fisher exact test. The level of significance was set at α = 0.05. The descriptive analysis and all statistical analyses were performed with IBM SPSS Statistics software, version 25.0 (IBM, Armonk, NY, USA).

## Results

By May 31, 2020, there were 2249 persons with RT-PCR-confirmed SARS-CoV-2 in Croatia. Among them, 46.0% were men, the median age was 51 years (minimum-maximum range: 0-98), and the median disease duration was 27 days (minimum-maximum range: 0-77). Hypertension was present in 35.5% of patients, circulatory system diseases excluding hypertension in 23.9%, diabetes in 10.9%, chronic lower respiratory diseases in 9.5%, cancer in 6.5%, ischemic heart disease in 5.4%, cerebrovascular diseases in 4.7%, cardiomyopathy in 4.4%, and other chronic obstructive pulmonary disease and chronic kidney disease both in 2.4%.

Patients' characteristics, including the prevalence of comorbidities and differences between survivors and non-survivors, are presented in Table 1. Patients who died were significantly older, had a higher prevalence of all diagnosed comorbidities, more frequently required mechanical ventilation support, and were more frequently hospitalized; however, their average disease duration was shorter and there were no differences in the case-fatality rate between men and women.

**Table 1:** Characteristics of patients with coronavirus disease-2019 regarding clinical outcome

	No. (%) of		
	non-survivors (N = 106)	survivors (N = 2143)	P
Age (years)*	82 (43-98)	50 (0-98)	<0.001
Sex			
men	46.2	46.0	0.965
women	53.8	54.0	
Comorbidity			
diabetes (E10-E14)	25.5	10.2	<0.001
hypertension (I10-I15)	70.8	33.8	<0.001
ischemic heart diseases (I20-I25)	18.9	4.7	<0.001
cerebrovascular diseases (I60-I69)	23.6	3.8	<0.001
cardiomyopathy (I42)	30.2	3.1	<0.001
diseases of the circulatory system, excluding hypertension (I00-I09 and I20-I99)	64.2	21.9	<0.001
chronic lower respiratory diseases (J40-J47)	24.5	8.7	<0.001
other chronic obstructive pulmonary disease (J44)	10.4	2.0	<0.001
cancer (C00-C97)	22.6	5.7	<0.001
chronic kidney disease (N18)	10.4	2.0	<0.001
Hospitalization	99.1	38.9	na
Mechanical ventilation	34.0	1.0	<0.001
Disease duration (days)*	13 (0-61)	27 (1-77)	<0.001

*Data are presented as median (minimum-maximum).

There were no differences with respect to the frequency of ischemic heart diseases and chronic kidney disease between patients who required mechanical ventilation support and those who did not. However, patients requiring mechanical ventilation support were more frequently men, on average older with a higher prevalence of all other investigated comorbidities, more frequently required hospitalization, and had longer disease duration compared with patients who did not require mechanical ventilation support (Table 2).

**Table 2:** Characteristics of patients with coronavirus disease-2019 with regard to mechanical ventilation support

	No. (%) of patients treated		
	with mechanical ventilation support (N = 57)	without mechanical ventilation support (N = 2192)	*P*
Age (years)*	70 (31-95)	51 (0-98)	<0.001
Sex			
men	59.6	45.7	
women	40.4	54.3	0.037
Comorbidity			
diabetes (E10-E14)	24.6	10.6	0.001
hypertension (I10-I15)	66.7	34.7	<0.001
ischemic heart diseases (I20-I25)	7.0	5.3	0.545
cerebrovascular diseases (I60-I69)	19.3	4.3	<0.001
cardiomyopathy (I42)	21.1	4.0	<0.001
diseases of the circulatory system excluding hypertension (I00-I09 and I20-I99)	56.1	23.0	<0.001
chronic lower respiratory diseases (J40-J47)	24.6	9.1	<0.001
other chronic obstructive pulmonary disease (J44)	8.8	2.2	0.001
cancer (C00-C97)	19.3	6.2	<0.001
chronic kidney disease (N18)	3.5	2.3	0.391
Hospitalization	100.0	40.2	na
Death	63.2	3.2	<0.001
Duration of disease (days)*	27 (1-77)	27 (0-72)	0.018

*Data are presented as median (minimum-maximum).

Hospitalization frequency, mechanical ventilation frequency, and case-fatality rates increased with patient age. The total hospitalization rate was 41.8%; age-specific hospitalization rates increased from 16.0% in 0-19 years age group to 100.0% in 90+ years age group. Mechanical ventilation support rate was 2.5%, ranging from 0.0% to 9.0%, with the highest rate in 70-79 age group. Case-fatality rates increased with age from 0.0% in 0-19 age group to 46.8% in 90+ age group, with a total case-fatality rate of 4.7% (Table 3).

**Table 3:** Hospitalization rates, mechanical ventilation rates, and case-fatality rates among coronavirus disease 2019 patients in Croatia with regard to sex and age

Age group	Hospitalization rates			Mechanical ventilation rates			Case-fatality rates		
	men	women	total	men	women	total	men	women	total
**0-19**	11.8	19.5	16.0	0.0	0.0	0.0	0.0	0.0	0.0
**20-29**	26.8	17.6	22.2	0.0	0.0	0.0	0.0	0.0	0.0
**30-39**	29.0	17.7	23.8	1.2	0.0	0.7	0.0	0.0	0.0
**40-49**	43.0	22.6	32.0	0.6	1.1	0.9	1.3	1.1	1.2
**50-59**	38.8	27.3	32.6	2.4	0.4	1.3	3.3	0.8	2.0
**60-69**	56.0	48.0	52.1	8.2	2.0	5.2	5.0	2.0	3.6
**70-79**	75.6	78.3	77.1	8.9	9.2	9.0	16.7	10.0	12.9
**80-89**	85.0	87.3	86.6	6.7	2.4	3.8	23.3	15.1	17.7
**90+**	100.0	100.0	100.0	16.7	7.3	8.5	50.0	46.3	46.8

## Discussion

The results of this study confirm the hypothesized association between the poor clinical outcomes of COVID-19 and pre-existing chronic somatic comorbidities, already shown by several previous studies. However, the underlying pathophysiological mechanisms contributing to adverse disease outcomes remain largely unknown (15). Additionally, previous meta-analyses also confirmed the correlation of pre-existing chronic diseases and poor COVID-19 prognosis (16,17). However, a better understanding of relevant pathophysiological mechanisms will offer new opportunities to develop more efficient and economical therapeutic options that would decrease the overall COVID-19 disease burden. Such research is also necessary in order to elucidate SARS-CoV-2-specific factors resulting in adverse disease outcomes, as opposed to factors related to other infectious disease causal agents (18).

COVID-19 poses a serious threat to global public health and a major burden to hospital systems (10). The incidence of COVID-19 in most Western countries has reached alarming rates, leading to a large number of patients requiring intensive care unit treatment, including the use of mechanical ventilation support, and generating additional costs to the already financially strained health care systems (19,20). In the absence of specific antiviral therapy, public health interventions aimed at precluding SARS-CoV-2 transmission still remain the most significant method of mitigating the medical and subsequent social and economic effects of the pandemic (21). The results of this study support the premise that SARS-CoV-2 vaccine should be readily available to the elderly population and patients suffering from chronic somatic diseases.

An earlier South Korean nation-wide cohort study reported that as many as 60% of the total number of COVID-19 patients may be asymptomatic at diagnosis, while more recently published reviews from various countries, including European ones, estimate the figure to be between 20% and 45% (22-24). Nevertheless, the estimation of the actual number of SARS-CoV-2-positive patients in a given population is challenging, and the results of studies on clinical and epidemiological characteristics of COVID-19 have to interpreted with caution. Greater availability of SARS-CoV-2 microbiological testing has immensely improved the estimations of incidence and prevalence rates of COVID-19, thus allowing for more valid assessments. It has also further contributed to contact tracing and other epidemiological interventions, proven to be of especial importance to the most vulnerable patient groups (25).

The limitations of this study are mainly related to data sources. Although databases are comprehensive and cover all patients in Croatia, thus enabling analyses on the national level, they do not contain information on the disease duration or staging, and on indicators that can influence the outcome independently of patient age. Furthermore, the ICD-10 codes pertaining to the patients' diagnoses may have in some cases been misattributed due to a lack of knowledge of the ICD-10 coding system or the misunderstanding of patients' symptoms at referral. The median age of COVID-19 patients in this study was higher than the average age of Croatian population, which partially explains the higher prevalence of chronic diseases and greater overall mortality. Moreover, the studied sample constitutes only a small fraction of the Croatian population, and future studies with larger patient samples should better address the infection spread patterns in the population.

Patients who were infected with COVID-19 and died during the first epidemic wave were significantly more likely to suffer pre-existing somatic comorbidities, such as circulatory system disease, diabetes, cancer, chronic lower respiratory, and kidney disease, while there were no differences in the outcome between the sexes. Patients requiring mechanical ventilation support more frequently suffered the investigated comorbidities, except ischemic heart disease and chronic kidney disease. Moreover, men required ventilator respiration support more often than women did. These results corroborate the findings of previous similar studies and call for further research into underlying biological mechanisms, an effort that could optimize the efficiency of preventive measures.
